# Antioxidant Properties of the Edible Basidiomycete *Armillaria mellea* in Submerged Cultures

**DOI:** 10.3390/ijms12106367

**Published:** 2011-09-26

**Authors:** Ming-Yeou Lung, Yu-Cheng Chang

**Affiliations:** Department of Chemical and Materials Engineering, Minghsin University of Science and Technology, Hsin Fong, Hsin Chu 304, Taiwan; E-Mail: moonriversnow@hotmail.com

**Keywords:** *Armillaria mellea*, submerged culture, antioxidant property, antioxidant components

## Abstract

Antioxidant components, ascorbic acid, total flavonoids and total phenols are produced effectively by *Armillaria mellea* submerged cultures. Dried mycelia and mycelia-free broths obtained by *A. mellea* submerged cultures are extracted with methanol and hot water and investigated for antioxidant properties. Methanolic extracts from dried mycelia (MEM) and mycelia-free broth (MEB) and hot water extracts from dried mycelia (HWEM) by *A. mellea* submerged cultures show good antioxidant properties as evidenced by low EC_50_ values (<10 mg/mL). Total flavonoid is mainly found in hot water extracts; however, total phenol is rich in methanol and hot water extracts from mycelia. Ascorbic acid and total phenol contents are well correlated with the reducing power and the scavenging effect on superoxide anions. Total flavonoid content is dependent on the antioxidant activity and the chelating effect on ferrous ions. Total antioxidant component contents are closely related to the antioxidant activity and the scavenging superoxide anion ability. Results confirm that extracts with good antioxidant properties from fermenting products by *A. mellea* are potential good substitutes for synthetic antioxidants and can be applied to antioxidant-related functional food and pharmaceutical industries.

## 1. Introduction

Active free radicals are derived from by-products of biological reactions or exogenous factors, and reactive oxygen species (ROS) are generated by unavoidable life consequences in normal aerobic metabolisms. Reactive oxygen species (ROS), including hydroxyl (OH^·^), superoxide anion radical (O_2_^·−^), nitric oxide (NO^·^), peroxyl (ROO^·^), alkoxyl (RO^·^), hydrogen peroxide (H_2_O_2_) and hypochlourous acid (HOCl) cause oxidative damages related to aging and many diseases, such as cancer, atherosclerosis, and rheumatoid arthritis [1]. However, the uncontrolled ROS production starts many human diseases such as cancer, atherosclerosis, reperfusion injury, hepatic injury, degenerative processes associated with aging through lipid peroxidation and inhibition of protein synthesis and so on. Almost all organisms may be protected by enzymes of superoxide dismutase (SOD), catalase (CAT) and peroxidase from ROS damages, but unbalanced mechanisms such as ageing, diseases and physiogical function deterioration frequently cause damages because of insufficient enzymes. Recently, many synthetic antioxidants such as butylated hydroxyanisole (BHA), butylated hydroxytoluene (BHT) and *tert*-butylhydroquinone (TBHQ) are often used to reduce the oxidative damages by these ROS [2], but are suspected to be responsible for the liver damage and carcinogenesis [3]. Consequently, it is essential to develop potent natural antioxidants with less toxicity to substitute synthetic antioxidants.

*Armillaria mellea*, also known as honey mushroom, belongs to an edible and medicinal mushroom of the Tricholomataceae family. *A. mellea* has a strong symbiotic relationship with *Gastrodia elata*, known as Tian Ma of the Orchidaceae family. It has been used as a traditional medicine in Asia to treat various human medical diseases such as headache, insomnia, neurasthenia, palsy, dizziness, numbness in limbs, infantile convulsion and microbial infectious diseases [4]. Several researchers have reported that some bioactive components from *A. mellea* have been isolated and characterized [5–10], and possess potential biological activities [4–6,10–12]. Among all bioactive components from *A. mellea*, phenolic compounds are especially important due to their potential application to protect human body from oxidant stress [13]. Submerged culture is an efficient method to produce from many mushrooms natural antioxidants such as phenols, carotenoids, flavonoids and phenolic acids [1,14]. Antioxidant activity is one of the most important bioactivities of various mushroom extracts. Antioxidant properties of various solvent extracts from mushroom submerged cultures investigated by many investigators are reported to be highly related to their total phenols and total flavonoid content. Up till now, there is relatively little information about antioxidant properties of the extracts from *A. mellea* submerged cultures. Therefore, attempts are focused on the exploration of novel natural antioxidants as possible substitutes for synthetic antioxidants by *A. mellea* submerged cultures.

This paper accordingly evaluates the antioxidant properties of methanol extracts from mycelia and filtrate and hot water extracts from mycelia by *A. mellea* submerged culture. Antioxidant properties are determined in various test systems including antioxidant activity by the conjugated diene method, reducing power, scavenging abilities on DPPH (2,2-diphenyl-1-picryl hydrazyl radical), chelating ability on ferrous ions and scavenging superoxide anion activity. Contents of potential antioxidant components in these extracts are also examined.

## 2. Results and Discussion

### 2.1. Extraction Yields from Mycelia and Broth

Extraction yields of three extracts from fermented products by *A. mellea* submerged cultures are summarized in Table 1. The yields of MEM, MEB and HWEM are 2.45, 4.16 and 3.89 g/10 g dry weight, respectively. The individual extract percentage yields for 10 g dry weight are 24.52%, 41.68% and 38.91% for MEM, MEB and HWEM. Extract percentage yield order of the three extracts are MEB > HWEM > MEM. For methanolic extracts, the yield of MEB is 1.7-fold higher than that of MEM. MEB seems to contain more methanol-soluble substances than mycelia. Yield of hot water extract of mycelia is apparently higher than that of methanolic extract. However, yields of extracts are not directly related to their antioxidant activities. Antioxidant activities of extracts are generally correlated with antioxidant component contents in extracts [1,15]. The yield (38.91%) of hot water extract from mycelia by *A. mellea* is superior to those from other mushrooms such as *Hypsizigus marmoreus* (26.37%) [16] and *Phellinus igniarius* (15.62%) [1]. In addition, yield of methanolic extract from mycelia-free broth by *A. mellea* is lower than that from *P. igniarius* [1]. Methanolic extract from mycelia by *A. mellea* has similar extraction yield (about 24%) with methanolic mycelia extracts from some other mushrooms such as *Grifola frondosa* (24.5%) and *Termitomyces albuminosus* (24.2%), but about 2-fold above that from *Morchella esculenta* (13.5%) [17]. Some bioactive components such as polyphenolics, triterpenoid, and steroids can be extracted by methanol or ethanol from mushrooms. Wong and Chye [14] reported that phenolic acid, lignans and flavonoids with –OH and –COOH functional groups are easily extracted by polar solvents. The extract yield variation from *A. mellea* submerged cultures is probably due to the differences in strains, culture conditions and harvest time.

### 2.2. Antioxidant Activity

**Figure 1:**
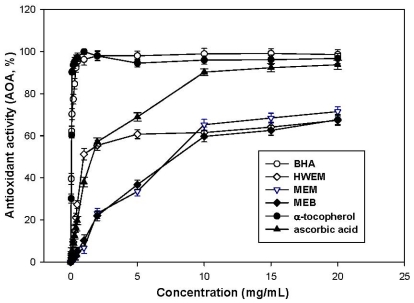
Antioxidant activities of MEM, MEB and HWEM from submerged culture of *Armillaria mellea*. Each value is expressed as mean ± standard deviation (*n* = 3).

Figure 1 shows that antioxidant activities of MEM, MEB and HWEM are marked and related to concentrations. Significant antioxidant activities are evident for all concentrations of all extracts. At 0.5–20 mg/mL, antioxidant activities are 4.3–71.6%, 5.2–67.6% and 27.3–67.3% for MEM, MEB and HWEM, respectively. At lower extract concentrations (0–10 mg/mL), HWEM possesses evidently higher antioxidant activities than MEM and MEB. Antioxidant activity of MEM is almost close to that of MEB. Above 10 mg/mL, however, similar antioxidant activity is found in MEM, MEB and HWEM. At 5 mg/mL, HWEM displays the highest antioxidant activity (60.7%) compared to MEM (33.5%) and MEB (36.8%). The antioxidant standards including BHA, α-tocopherol and ascorbic acid exhibit apparently higher antioxidant activities of 98.0%, 94.5% and 68.9% at 5 mg/mL, respectively. For methanolic extracts, Lung *et al*. [1] found that antioxidant activities of methanolic extracts from *P. ignirius* mycelia and broth were 15.2%–68.3% and 18.3%–75.4% at 0.5–20 mg/mL. The methanolic extracts from *Ganoderma tsugae* and *Agrocybe cylindracea* mycelia reveal antioxidant activities of 21.5%–67.4% and 7.6%–19.3% at 0.5–20 mg/mL [18]. Compared with these results, antioxidant activity of MEM is comparable to those of methanolic extracts from *P. ignirius* and *G. tsugae* mycelia, but is better than that of *A. cylindracea* mycelia. Moreover, MEB is slightly weaker in antioxidant activity than methanolic extract from *P. ignirius* broth. With regard to hot water extracts, Lee *et al*. [16] noted that the antioxidant activity of hot water extract from *H. marmoreus* mycelia was 32.6% at 5 mg/mL. In addition, hot water extract from *P. ignirius* mycelia shows an antioxidant activity of 28.2% at 5 mg/mL [1]. At the same concentration, hot water extract from *A. mellea* submerged culture seems to more potent than *P. ignirius* and *H. marmoreus* mycelia in antioxidant activity. These results clearly indicate that the extracts from *A. mellea* submerged cultures have potential antioxidant activities.

### 2.3. Reducing Power

Reducing properties of antioxidants are generally associated with reductones which can utilize antioxidation to break free radical chains by donating hydrogen atoms [19] and can also react with certain precursors of peroxide to prevent peroxide formation. Therefore, the reducing capacity of a compound may serve as a significant antioxidant potential indicator [20]. Efficiencies of certain antioxidants have been reported to be associated with their reducing powers.

**Figure 2:**
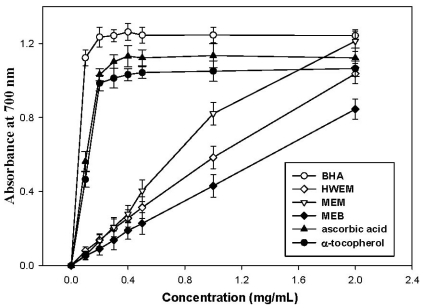
Reducing power of MEM, MEB and HWEM from submerged culture of *Armillaria mellea*. Each value is expressed as mean ± standard deviation (*n* = 3).

All extracts tested showed high reducing powers, which correlate well with increasing concentrations (Figure 2). MEM, MEB and HWEM at 2.0 mg/mL exhibit good reducing powers of 1.21, 0.85 and 1.04, respectively. Between 0 to 2 mg/mL, the order for reducing powers is MEM > HWEM > MEB. At 0.2 mg/mL, reducing power sequences of BHA, α-tocopherol and ascorbic acid are 1.24, 0.99 and 1.03, respectively, indicating that these commercial antioxidants reveal higher reducing abilities compared to the extracts from *A. mellea* submerged cultures. Similar results are found in other mushroom extracts reported earlier [1,14,16]. Mau *et al*. [17] noted that the reducing power of the methanol extract from *M. esculenta* mycelia is 0.97 at 25 mg/mL. In addition, methanol extracts from *G. frondosa* and *T. albuminosus* mycelia show reducing powers of 0.30 and 0.37 at 5 mg/mL [17]. Apparently, the reducing power of MEM is higher than those of the extracts from *M. esculenta*, *G. frondosa* and *T. albuminosus*. Lung *et al*. [1] reported that the reducing power of the extract from mycelia-free broth of *P. igniarius* was 0.40 at 5 mg/mL. Compared with this result, MEB is more effective in reducing power. Lee *et al*. [16] observed that hot water extract from *H. marmoreus* mycelia was about 0.75 at 5 mg/mL, which was below that of *A. mellea* mycelia used in this investigation. The above results indicate that all extracts from *A. mellea* submerged culture exhibit excellent reducing powers to donate electrons to terminate radical chain reactions.

### 2.4. DPPH Radical-Scavenging Effect

**Figure 3:**
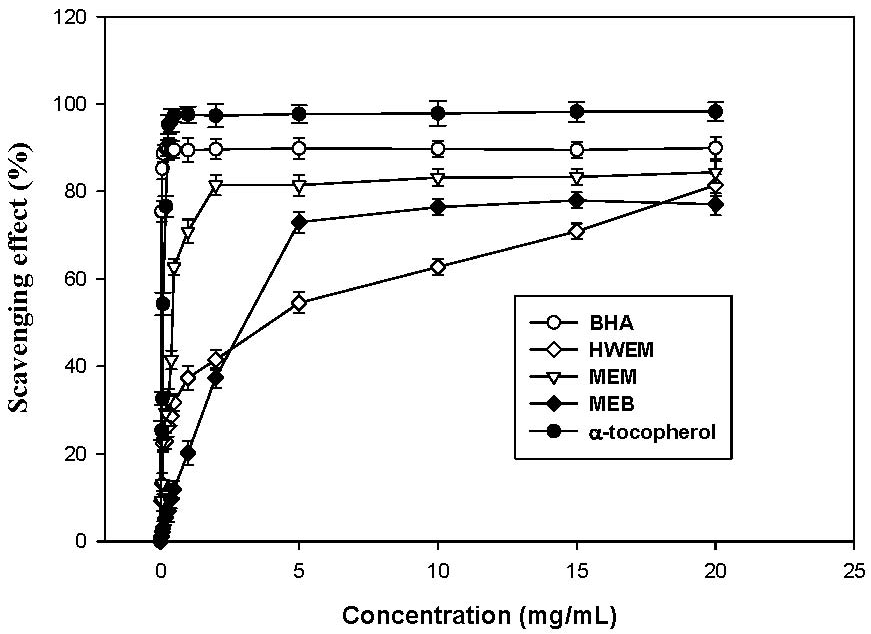
Scavenging effect of MEM, MEB and HWEM from submerged culture of *Armillaria mellea* on 2,2-diphenyl-1-picrylhydrazyl radical. Each value is expressed as mean ± standard deviation (*n* = 3).

Figure 3 shows the scavenging ability on DPPH radicals of extracts from *A. mellea* submerged cultures. From 0.0 to 5 mg/mL, the scavenging ability on DPPH radical of MEM is much higher than those of MEB and HWEM. The higher the concentration, the higher the scavenging ability of extracts. However, scavenging abilities of extracts are lower than those of BHA and α-tocopherol. Similar results have been reported for other mushrooms [1,16,17].

Mau *et al*. [17] reported that scavenging effects of three methanol extracts at 10 mg/mL on DPPH radicals were 78.8%, 79.4% and 94.1% for *T. albuminosus*, *G. frondosa* and *M. esculenta* mycelia, respectively. The scavenging DPPH radical ability (83.2%) of the methanol extract at 10 mg/mL from *A. mellea* submerged cultures is comparable to those of *T. albuminosus* and *G. frondosa* mycelia but less effective than that of *M. esculenta* mycelia. Lung *et al*. [1] pointed out that the methanol extract from mycelia-free broth by *P. igniarius* submerged culture exhibited a scavenging DPPH radical capacity of 30.0% at 10 mg/mL, which was obviously lower than that (76.4%) of *A. mellea* submerged cultures in this research. Scavenging DPPH radical abilities of hot water extracts from mycelia are 81.8% and 43.1% at 10 mg/mL for *H. marmoreus* [16] and *P. igniarius* [1]. Hot water extracts from mycelia of *A. mellea* submerged cultures are stronger in scavenging DPPH radical capacity (62.7%) than *P. igniarius*, but weaker than *H. marmoreus*. These results suggest that extracts from *A. mellea* submerged cultures reveal a considerably high scavenging ability on DPPH radicals.

Free radicals are deleterious to cellular components and cellular functions. Scavenging free radicals may inhibit lipid oxidation. Several researchers have shown that various extracts from mushrooms are good free radical inhibitors or scavengers, acting possibly as primary effective antioxidants against free radicals [1,14,16,17]. Consequently, extracts from *A. mellea* submerged cultures might be good free radical inhibitors or scavengers to protect the human body from oxidative damages.

### 2.5. Chelating Abilities on Ferrous Ions

Transition metals have been reported as catalysts to initiate radical formation. Chelating agents stabilize transition metals in living systems, inhibit free radical generation and accordingly reduce induced damages by free radicals. In addition, metal irons can combine proteins to form coenzymes which accelerate bio-reactions in cells. Ferrous ions can stimulate lipid peroxidation known as Fenton reaction, and also accelerate peroxidation to decompose lipid hydroperoxides into peroxyl and alkoxyl radicals [21]. The development of potential chelating agents from natural mushrooms thus provides an effective way to protect human beings from free radical damages.

**Figure 4:**
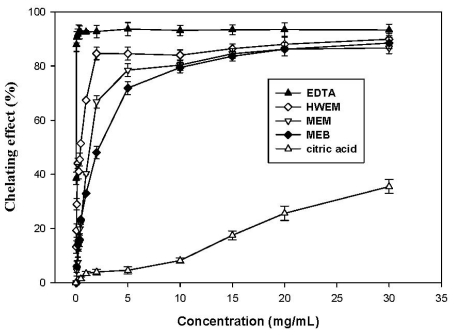
Chelating effect of MEM, MEB and HWEM from submerged culture of *Armillaria mellea* on ferrous ions. Each value is expressed as mean ± standard deviation (*n* = 3).

As shown in Figure 4, all extracts exhibit different dose-dependent chelating abilities on ferrous ions. The Fe^2+^ chelating activity sequence is HWEM > MEM > MEB. HWEM and MEM at 2.0 mg/mL reveal good chelating potencies of 84.6% and 66.8%, respectively, which are much higher than MEB (48.0%). At 5.0 mg/mL, HWEM, MEM and MEB chelate ferrous ions by 84.5%, 78.5% and 71.9%, respectively. However, EDTA shows the strongest chelating ability of 90.6% at a lower concentration of 0.1 mg/mL. In contrast, citric acid displays a considerably weak chelating capacity of 4.6% at 5.0 mg/mL. Chelating activities of HWEM and MEM above 20 mg/mL are comparable to that of EDTA, but significantly higher than that of citric acid at the concentration between 0 and 30 mg/mL. Tsai [18] noted that methanolic extracts from mycelia of *G. tsugae* and *A. cylindracea* chelated ferrous ions by 80.2% and by 84.6% at 5 mg/mL, respectively. Fe^2+^ chelating activity of methanolic extract from *P. igniarius* mycelia was 60.3% at 5 mg/mL as reported by Lung *et al*. (2010) [1]. Chelating activity of MEM in this study on ferrous ions is comparable to those of *G. tsugae* and *A. cylindracea*, and more effective than that of *P. igniarius*. Lung *et al*. [1] pointed out that methanolic extracts from mycelia-free broth of *P. igniarius* showed a chelating activity of 79.1% at 5 mg/mL, which was slightly higher than MEB at the same concentration. For hot water extract from mycelia, chelating capacity of *H. marmoreus* is 29.1% at 5 mg/mL [16]. Fe^2+^ chelating ability of hot water extract from *P. igniarius* mycelia reaches 44.6% at 5 mg/mL [1]. Comparision among these results conclude that the chelating activity (84.5%) of HWEM is higher than those of *H. marmoreus* and *P. igniarius* and also indicate that extracts from *A. mellea* submerged cultures show potent chelating abilities on ferrous ions and may be best employed as chelating agents to inhibit lipid peroxidation and free radicals damages.

### 2.6. Scavenging Effect on Superoxide Anion

**Figure 5:**
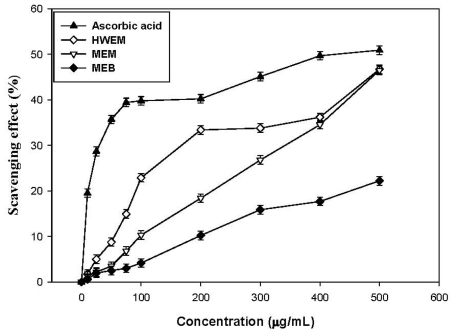
Scavenging effects of MEM, MEB and HWEM from submerged culture of *Armillaria mellea* on superoxide anion. Each value is expressed as mean ± standard deviation (*n* = 3).

Superoxide is a relatively weak oxidant and can decompose to form stronger reactive oxidative species, such as singlet oxygen and hydroxyl radicals with strong oxidative and oleophilic abilities [22]. Superoxide, one of the precursors of singlet oxygen or hydroxyl radicals, can indirectly induce lipid peroxidation by hydroxyl radicals derived from H_2_O_2_ formation. Moreover, superoxide radical and its derivatives may bring damages to DNA and cell membrane and induce many pathological incidents such as arthritis and Alzheimer’s disease [23,24]. Therefore, it is exceptionally important to characterize the superoxide radical scavenging potential of different antioxidants from various natural sources such as mushrooms. As shown in Figure 5, extracts dose-dependently exhibit a superoxide radical scavenging ability at all concentrations from 0 to 500 μg/mL. HWEM is found to be the most effective superoxide radical scavenger of all the extracts. At 200 μg/mL, the superoxide radical scavenging abilities are 18.4%, 10.2% and 33.4% for MEM, MEB and HWEM, respectively, where activities can be ranked as HWEM > MEM > MEB. However, ascorbic acid at 200 μg/mL reveals higher superoxide radical scavenging ability (40.2%) compared to all the extracts. Lung *et al*. [1] reported that MEM, MEB and HWEM from *P. igniarius* submerged cultures showed superoxide radical scavenging activities of 23.5%, 8.2%, and 26.5% at 200 μg/mL. Apparently, the superoxide radical scavenging abilities of all the extracts from *A. mellea* submerged cultures herein are comparable to those of *P. igniarius* as reported earlier by Lung *et al*. [1]. These results clearly indicate that antioxidant activities of all the extracts are related to scavenging superoxide radical abilities, which can help to prevent ameliorate oxidative damages.

### 2.7. EC_50_ Values in Antioxidant Properties

Antioxidant properties assayed herein were summarized in Table 2 with results normalized and expressed as EC_50_ values in the unit of mg/mL for comparison. Lower EC_50_ values indicate higher efficiency in antioxidant properties. Generally, extracts are good in antioxidant properties when EC_50_ values are below 10 mg/mL [17]. For antioxidant activity by the conjugated diene method, EC_50_ values of the extracts decreases in the order of MEB (7.83 mg/mL) > MEM (7.42 mg/mL) > HWEM (5.51 mg/mL), where all extracts show good antioxidant activities due to the EC_50_ values below 10 mg/mL. Hot water extracts of mycelia with lower EC_50_ values are more effective in antioxidant activity than methanol extracts. BHA, ascorbic acid and α-tocopherol present strong antioxidant activities with significantly lower levels of 0.061, 1.621 and 0.067 mg/mL, respectively. EC_50_ values of all extracts for reducing power are below 1.5 mg/mL and descend in the order of MEB (1.34 mg/mL) > HWEM (0.91 mg/mL) > MEM (0.73 mg/mL), indicating that MEM is the best of all. The standards, BHA, ascorbic acid and α-tocopherol reveal strong reducing powers owing to the EC_50_ values below 0.2 mg/mL. EC_50_ values for scavenging ability on DPPH radicals are 2.96, 8.62 and 7.88 mg/mL for MEM, MEB and HWEM, respectively, and resulted order is MEB > HWEM > MEM with MEM being the most effective DPPH radical scavenger of all the extracts. BHA and α-tocopherol exhibit considerably lower EC_50_ value in DPPH radical-scavenging activity (EC_50_ < 0.1mg/mL). For chelating ability on ferrous ions, EC_50_ values of MEM, MEB and HWEM reach 5.98, 4.35 and 1.81 mg/mL, respectively and HWEM shows the strongest chelating activity because of the lowest EC_50_ value. With EC_50_ apparently above EDTA (0.059 mg/mL) but below citric acid (42.64 mg/mL), the extracts have potent chelating abilities. EC_50_ values for scavenging activity on superoxide radicals are 0.55, 1.11 and 0.51 mg/mL for MEM, MEB and HWEM, respectively. EC_50_ level of MEM is almost equal to that of HWEM and is higher than that of ascorbic acid (0.454 mg/mL) (p < 0.05). In general, since all EC_50_ values of the extracts for antioxidant properties analyzed in this study are below 10 mg/mL, the extracts from *A. mellea* submerged cultures have potential antioxidant abilities and may serve as a novel antioxidant source to be applied in food and pharmaceutical industries.

### 2.8. Antioxidant Components

Ascorbic acid is one of the major antioxidants and has diverse physiological roles to act as a scavenger for free radicals and/or as an electron donor for ascorbate peroxidase (APX) to scavenge hydrogen peroxide involved in the ascorbate–glutathione cycle [25]. Ascorbic acid can interact directly with superoxide radical and hydrogen radical in plasma and assist α-tocopherol to inhibit lipid peroxidation by recycling the tocopherol radical [26]. In this research, similar ascorbic acid contents (about 3.7 mg/g extract) are found in the three extracts (Table 3), which are apparently higher than those in the other mushroom extracts such as *Pleurotus ostreatus* (0.25 mg/g extract) as reported by Jayakumar *et al*. [27]. Ascorbic acid contents of the extracts are correlated with EC_50_ values of reducing power and scavenging effect on superoxide anions with relevant correlation coefficients (*R*^2^) of 0.998 and 0.853.

Carotenoids have been reported to act as radical scavengers due to the extensive system of conjugated double bonds in their molecule, and β-carotene is an excellent scavenger of singlet oxygen [28]. As shown in Table 3, β-carotene appears only in hot water extract from mycelia with content of 0.05 mg/g extract, higher than those of methanol extracts from other seven edible wild mushrooms [29].

Phenolic compounds, such as flavonoids and phenolic acids, are considered to be major contributors to the antioxidant capacity of the extracts from mushroom. These antioxidants also possess diverse biological activities as anti-inflammatory, anti-atherosclerotic and anticarcinogenic activities, which may be related to their antioxidant activity [1,16]. In this study, hot water extracts from mycelia by *A. mellea* submerged cultures are highly rich in total flavonoid as compared to methanol extracts from mycelia and broth (Table 3). Total flavonoid contents of MEM, MEB and HWEM are 6.80, 7.39 and 19.2 mg/g extract, respectively. There is an excellent correction between total flavonoid content and EC_50_ values of antioxidant activity and chelating effect on ferrous ions. The corresponding coefficients (*R*^2^) are 0.957 and 0.853, respectively.

Polyphenolic compounds seem to be important for lipid oxidation stabilization and are associated with antioxidant activity [1,30]. In this work, total phenols are the major antioxidant components in hot water and methanol extracts from mycelia. The order of total phenol contents in the extracts is HWEM (30.9 mg/g) > MEM (27.1 mg/g) > MEB (11.9 mg/g). Total phenol content correlates well with EC_50_ values for reducing power and scavenging effect on superoxide anion with correlation coefficients (*R*^2^) of 0.786 and 0.983, respectively. In addition, total antioxidant component contents (Ascorbic acid + total flavonoid + β-carotene + Total phenols) of the three extracts are 37.7, 22.9 and 53.9 mg/g for MEM, MEB and HWEM, respectively. Total antioxidant component contents are correlated well with EC_50_ values for antioxidant activity (*R*^2^ = 0.894) and scavenging superoxide anion ability (*R*^2^ = 0.789).

Antioxidant activity of phenolics is mainly due to the redox properties which allow them to act as reducing agents, hydrogen donators and single oxygen quenchers [21]. Several researchers have recently presented a correlation between total phenolic content and antioxidant activity in mushroom extracts [1,2,15]. The correlation for reducing power and scavenging effect on superoxide anion is built by taking into consideration that various phenolic contents are present in fermented *A. mellea* mushroom extracts. Consequently, total phenols in the three extracts from *A. mellea* submerged cultures might be the possible major components contributing to their antioxidant properties. Ascorbic acid and total flavonoid may play an important role on the antioxidant properties of the extracts. It is reasonable to expect that some other specific active components might be involved in these properties and further isolation, fractionation and identification for other potential bioactive components from these extracts are looked forward to.

## 3. Experimental Section

### 3.1. Chemicals

Potassium ferricyanide, trichloroacetic acid, butylated hydroxyanisole (BHA), α-tocopherol and ascorbic acid, 2,2-diphenyl-1-picryl hydrazyl radical (DPPH), ferrozine, phenazine methosulfate (PMS), nitro blue tetrazolium (NBT), 2,6-dichloroindophenol, L-ascorbic acid standard, dichloromethane, β-carotene standard, quercetin, Folin-Ciocalteau reagents and gallic acid were purchased from Sigma Chemical Co. (St. Louis, MO, USA). NaNO_2_, AlCl_3_·6H_2_O, phosphate buffer, ethylenediaminetetraacetic acid (EDTA), ferric chloride and citric acid were from Merck & Co., Inc. (Darmstadt, Germany), metaphosphoric acid from Kanto Chemical Co., Inc. (Japan) and pyrogallol from Riedel-de Haen (Germany). Acetonitrile, ethanol, n-hexane and methanol were supplied by J.T. Baker, Mallinckrodt Baker, Inc. (Phillipsburg, NJ, USA). All chemicals and reagents used were analytical grade.

### 3.2. Mushroom Mycelia and Broth

*Armillaria mellea* BCRC 36362 was obtained from Bioresources Collection and Research Center in Hsinchu, Taiwan. The culture was maintained in a solid culture medium with the following compositions (g/L): malt extract, 20; glucose, 40; peptone, 1 and agar, 20. The sub-culture was conducted by transferring grown mycelia to a fresh nutrient agar medium every month. The three-week-old cells grown on the media agar plate were collected with 25 mL sterilized water mixed by mycelia. 20 mL collected mycelia were then transferred to 250 mL seed culture flasks containing 50 mL culture medium (g/L) composed of PDB (potato dextrose broth), 24; thiamine, 0.01; KH_2_PO_4_, 1.5 and MgSO_4_, 0.75. The seed culture was incubated at 28 °C on a rotary shaker at 125 rpm for 7 days.

*A. mellea* submerged culture products used in this study were obtained in a 5-L stirred tank bioreactor culture. The fermentation of *A. mellea* proceeded in a 5-L stirred tank bioreactors filled with 3 L culture medium and 5% (v/v) inoculums derived from seed cultures. The culture medium in the bioreactor was composed of 40.0 g/L glucose, 3.0 g/L yeast extract, 4.0 g/L KH_2_PO_4_ and 2.0 g/L MgSO_4_. The stirred tank bioreactor culture was operated at 22 °C, 1vvm (volume of aeration per volume of bioreactor per minute) aeration rate, 150 rpm agitation speed and controlled pH 4.0 for 14 days. The pH of culture medium was automatically controlled by adding 1 N HCl or 1 N NaOH. Mycelia were separated from fermented broth by centrifugation (4 °C, 8000 × g for 15 min), then washed with distilled water and finally freeze-dried to powders. Biomass concentration was determined in dry weight per unit volume. The mycelia concentration of 13.25 g/L was obtained for 14 days fermentation.

### 3.3. Preparation of Hot Water Extracts from Mycelia

Mixing and shaking 10 g mycelia powders with 100 °C distilled water in 250 mL flasks for 1 h at 150 rpm and 30 °C to prepare mycelial extracts. Extracts were filtered with Whatman No. 1 paper. Each extraction was repeated twice and the combined filtrates were concentrated under vacuum and freeze-dried to powders. Dried powders of mycelial extracts were prepared for various concentrations between 0.1 mg/mL and 20 mg/mL by adding distilled water for further use.

### 3.4. Preparation of Methanolic Extracts from Mycelia

Total of 10 g dried mycelia powders were extracted by mixing 100 mL methanol at 25 °C and 150 rpm for 24 h, and then filtered through Whatman No. 1 paper. The extraction was repeated and the combined methanolic extracts were rotary evaporated at 40 °C to dryness. Dried extracts were redissolved in methanol to various concentrations from 0.1 to 20 mg/mL for further use.

### 3.5. Preparation of Methanolic Extracts from Broth

Mycelia-free supernatants were collected by centrifuging *A. mellea* culture broth at 4 °C under 8000 × *g* for 10 min and then rotary evaporated at 40 °C to dryness. The 10 g dried supernatants were extracted by stirring with 100 mL methanol in a 250 mL flask and shaken for 24 h at 150 rpm and 25 °C. The extract was filtered through Whatman No. 1 paper. The extraction was repeated and the combined methanolic extracts were rotary evaporated at 40 °C to dryness. Dried extracts were mixed with methanol to various concentrations between 0.1 and 20 mg/mL for further use.

### 3.6. Antioxidant Activity

The antioxidant activity assay was examined by the conjugate diene method [31]. Each extract sample (0–20 mg/mL) of 0.5 mL was mixed with 2.5 mL 0.01 M linoleic acid emulsion in 0.2 M phosphate buffer (pH 6.5), and then placed in darkness at 37 °C to accelerate oxidation. After incubation for 15 h, 6 mL of 60% methanol in deionized water was added to the mixture, and the absorbance of the mixture solution was measured at 234 nm in a spectrophotometer (JASCO V-530, Japan). The antioxidant activity (AOA) was calculated with the following equation: AOA (%) = [(Δ*A*_234_ of control − Δ*A*_234_ of sample)/Δ*A*_234_ of control] × 100. AOA value of 100% corresponds to the strongest antioxidant activity. EC_50_ value in mg extract/mL expresses the effective concentration at which the antioxidant activity is 50% and is obtained by linear regression interpolation. Butylated hydroxyanisole (BHA), α-tocopherol, and ascorbic acid were used for comparison. Each value was presented as mean ± standard deviation (*n* = 3) of triplicate measurements.

### 3.7. Reducing Power

The reducing power was determined with the method described by Oyaizu [32]. Each extract sample (0–20 mg/mL) in methanol and deionized water (2.5 mL) was mixed with 2.5 mL 200 mM sodium phosphate buffer at pH 6.6 and 2.5 mL of 1% (w/v) potassium ferricyanide, and the mixture was incubated at 50 °C for 20 min. After 2.5 mL of 10% (w/v) trichloroacetic acid was added, the mixture was centrifuged at 200 × g for 10 min. The upper layer (5 mL) was mixed with 5 mL of deionized water and 1 mL of 0.1% (w/v) ferric chloride, and the absorbance was measured spectrophotometrically at 700 nm. EC_50_ value in mg extract/mL expresses the effective concentration at which the absorbance is 0.5 in the reducing power assay and is interpolated by linear regression. Ascorbic acid and α-tocopherol were used as standards. Data were presented as mean ± standard deviation (*n* = 3) of triplicate measurements.

### 3.8. DPPH Radical Scavenging Activity

A total of 4 mL extract samples of various concentrations (0–20 mg/mL) were mixed with 1 mL methanolic solution containing 1 mM DPPH radicals and resulted in a final concentration of 0.2 mM DPPH radicals. The mixture was shaken vigorously and left to stand for 30 min in darkness. DPPH radical reduction was determined by measuring the absorbance at 517 nm against a blank [33]. The scavenging ability was expressed as followed: [(Δ*A*_517_ of control −Δ*A*_517_ of sample)/Δ*A*_517_ of control] × 100. EC_50_ value in mg extract/mL expresses the effective concentration at which DPPH radicals are 50% and are obtained by linear regression interpolation. BHA, α-tocopherol and ascorbic acid were used as standards. Data were expressed by triplicate measurement with standard deviation.

### 3.9. Chelating Effects on Ferrous Ions

Chelating ability was examined according to the method of Dinis *et al*. [34]. Each extract sample (0–20 mg/mL, 1 mL) was mixed with 3.7 mL methanol and 0.1 mL 2 mM ferrous chloride. The mixture was then reacted with ferrozine (0.2 mL, 5 mM) for 10 min. Each value was expressed by triplicate measurement with standard deviation. With the absorbance reading at 562 nm (A_562_), chelating activities on ferrous ions were calculated by the following equation: Chelating effect (%) = [1 – (Δ*A*_562_ of sample)/(Δ*A*_562_ of control)] × 100%. A lower absorbance indicates a higher chelating power. EC_50_ value in mg extract/mL expresses the effective concentration at which ferrous ions are chelated by 50% and are obtained by linear regression interpolation. Ethylenediaminetetraacetic acid (EDTA) and citric acid were used as standards. The assay was performed in three duplicates and expressed as mean ± standard deviation (*n* = 3).

### 3.10. Scavenging Effects on Superoxide Anions

Scavenging capacity of extract samples on superoxide anions was assayed by the method of Robak *et al*. [35], in which reduction of nitro blue tetrazolium (NBT) was measured and superoxide anions were generated in the PMS-NADH system. Identical volumes of the sample, 30 μM phenazine methosulfate (PMS), 338 μM dihydronicotineamidadenine dinucleotide (NADH) and 72 μM NBT in 0.1 M phosphate buffer of pH 7.4 were mixed and incubated for reaction at the ambient temperature for 5 min. The absorbance was measured at 560 nm against blank samples. The scavenging capability to superoxide radicals was calculated as followed: [(Δ*A*_560_ of control −Δ*A*_560_ of sample)/Δ*A*_560_ of control] × 100. EC_50_ value in mg extract/mL expresses the effective concentration at which the scavenging superoxide anion activity is 50% and is interpolated by linear regression. Ascorbic acid was used for comparison. The results were expressed as mean ± standard deviation by triplicate measurement.

### 3.11. Determination of Antioxidant Components

Ascorbic acid was measured by the method of Klein and Perry [36] with minor modification. 20 mg dried methanol and water extracts from mycelia and mycelia-free broth by submerged culture of *A. mellea* were extracted with 10 mL of 1% metaphosphoric acid for 45 min at room temperature and then filtered through Whatman No. 1 paper. The absorbance of the mixture of 1 mL filtrates and 9 mL 2,6-dichloroindophenol was measured at 515 nm. The content of ascorbic acid depended on the calibration curve of L-ascorbic acid standard.

β-Carotene was determined by the method of Rundhaug *et al*. [37] with minor modification. Dried methanolic and water extracts from submerged culture of *A. mellea* were extracted with 1% pyrogallol solution in 10 mL methanol/dichloromethane (1:1, v/v) for 45 min at room temperature and filtered through Whatman No. 1 paper. The filtrate was finally adjusted to 10 mL with the same solution and then filtered through a 0.45 μM CA (cellulose acetate) filter before injected to high-performance liquid chromatograph (HPLC) to assay β-carotene content.

HPLC system (600E, Waters, Milford, MA, USA) is equipped with a Prodigy 5 ODS-2 column (250 × 4.6 mm, 5 μm, Phenomenex Inc., Torrance, CA, USA) and a model 2410 UV detector. All data were processed by Millennium software (Milford, MA, USA). The flow rate of the mobile phase of acetone/methanol/acetonitrile in the ratio of 1:2:2 (v/v/v) was 0.7 mL/min and the detecting wavelength by UV detector was 470 nm. β-carotene standards of various concentrations from 0 to 1000 mg/mL were used to construct a calibration curve.

Total flavonoid content was determined by the colorimetric method of Bao *et al*. [38] with minor modification. The 0.5 mL aliquots of appropriately diluted extracts or standard solutions were pipetted into 15 mL polypropylene conical tubes containing 2 mL double distilled H_2_O and mixed with 0.15 mL 5% NaNO_2_. After 5 min, 0.15 mL 10% AlCl_3_·6H_2_O solution was added and the mixture was allowed to stand for another 5 min, and then 1 mL 1 M NaOH was added to it. The reaction solution was well mixed and kept for 15 min. Absorbance of the mixture was examined at 415 nm. Total flavonoid content was calculated with the standard quercetin curve, and expressed in mg quercetin equivalent per gram of dry weight.

Total phenols were determined by the method of Taga *et al*. [39] with minor modification. 20 mg dried methanolic and water extracts from the submerged culture of *A. mellea* were dissolved in a 5 mL 1.3% methanol/deionized water solution (1.5:1, v/v), and 100 μL mixture was mixed with 2 mL 2% aqueous sodium carbonate solution. 100 μL 50% Folin-Ciocalteau reagents were added into the mixture 3 min later. After standing for 30 min, the mixture absorbance was determined at 750 nm. The total phenol content was calculated with the calibration curve of gallic acid.

### 3.12. Statistical Analysis

Experimental results were mean ± SD of three measurements. Data collected were subjected to an analysis of variance by SAS for Windows V8. One-way analysis of variance (ANOVA) and T tests (LSD) multiple comparisons were carried out to detect significant difference (*p* < 0.05) between the mean values that had more than two groups.

## 4. Conclusions

Low EC_50_ values of the investigation results evidence that methanolic extracts from mycelia (MEM) and broth (MEB) and hot water extracts from mycelia (HWEM) by *A. mellea* submerged cultures possess noticeable antioxidant properties. Total flavonoid is mainly present in hot water extracts and total phenol is rich in methanol and hot water extracts from mycelia. Ascorbic acid and total phenol contents are correlated well with the reducing power and the scavenging effect on superoxide anions. Total flavonoid content is dependent on the antioxidant activity and the chelating effect on ferrous ions. Total antioxidant component contents are closely related to the antioxidant activity and the scavenging superoxide anion ability. Extracts with powerful antioxidant properties from fermenting products by *A. mellea* are of great potential to antioxidant-related functional food and pharmaceutical industries as confirmed by the research.

## Figures and Tables

**Table 1:** Extraction yield of MEM, MEB, and HWEM from submerged cultures of *Armillaria mellea*.

	Yield (g/10 g dry weight)	Extraction % (w/w)
MEM	2.45 ± 0.025 C	24.52 ± 0.25 C
MEB	4.16 ± 0.032 A	41.68 ± 0.32 A
HWEM	3.89 ± 0.024 B	38.91 ± 0.24 B

Each value is expressed as mean ± standard deviation (*n* = 3).

A,B,C Within a column, means with different letters present significantly different (*p* < 0.05).

**Table 2:** EC_50_ values of MEM, MEB, and HWEM from submerged cultures of *Armillaria mellea*.

	EC_50_ (mg/mL)							
	
	Samples			Standards				
		
	MEM	MEB	HWEM	BHA	Ascorbic acid	α-tocopherol	EDTA	Citric acid
Antioxidant activity	7.42±0.11^B^	7.83±0.15^A^	5.51±0.19^C^	0.061±0.003^E^	1.621±0.082^D^	0.067±0.002^E^	-	-
Reducing power	0.73±0.03^C^	1.34±0.04^A^	0.91±0.04^B^	0.045±0.003^D^	0.094±0.005^D^	0.103±0.022^D^	-	-
Scavenging effect on	DPPH radicals	2.96±0.15^C^	8.62±0.21^A^	7.88±0.17^B^	0.033±0.004^D^	-	0.099±0.012^D^	-
Chelating effect on ferrous ions	5.98±0.21^B^	4.35±0.07^BC^	1.81±0.09^CD^	-	-	-	0.059±0.005^D^	42.64±2.58^A^
Scavenging effect on superoxide anion	0.55±0.02^B^	1.11±0.03^A^	0.51±0.01^BC^	-	0.454±0.03^C^	-	-	-

Each value is expressed as mean±standard deviation (*n* = 3);

A–E Within a row, means with different letters present significantly different (*p* < 0.05); EC_50_ value: the effective concentration where the antioxidant activity was 50%; the absorbance was 0.5 for reducing power; the DPPH radicals were scavenged by 50%; the ferrous ions were chelated by 50%; and the superoxide anion were scavenged by 50%, respectively; EC_50_ value was obtained by linear regression interpolation; BHA: Butylated hydroxyanisole; EDTA: Ethylenediaminetetraacetic acid.

**Table 3:** Contents of main antioxidant components from MEM, MEB, and HWEM from submerged cultures of *Armillaria mellea*.

Compound	MEM (mg/g extract )	MEB (mg/g extract)	HWEM (mg/g extract)
Ascorbic acid	3.78 ± 0.04 A	3.60 ± 0.12 A	3.72 ± 0.04 A
β-carotene	nd	nd	0.05 ± 0.01
Total flavonoid	6.80 ± 0.12 C	7.39 ± 0.15 B	19.2 ± 0.21 A
Total phenols	27.1 ± 0.21 B	11.9 ± 0.11 C	30.9 ± 0.17 A

Each value is expressed as mean ± standard deviation (*n* = 3).

A,B,C Within a row, means with different letters present significantly different (*p* < 0.05). nd: not detected.

## Abbreviations

BCRC: Bioresources Collection and Research Center in Hsinchu, Taiwan
BHA: butylated hydroxyanisole
DPPH: 2,2-diphenyl-1-picryl hydrazyl radical
EC_50_: the effective concentration where the antioxidant property is 50%
EDTA: ethylenediaminetetraacetic acid
HWEM: hot water extracts from dried mycelia by *A. mellea* submerged cultures
MEB: methanolic extracts from mycelia-free broth by *A. mellea* submerged cultures
MEM: methanolic extracts from dried mycelia by *A. mellea* submerged cultures
NBT: nitro blue tetrazolium
PDB: potato dextrose broth
ROS: reactive oxygen species
PMS: ferrozine, phenazine methosulfate
